# The role of standard non-ECG gated chest CT in cardiac assessment: design and rationale of the *Cardiac Pathologies in standard chest CT* (CaPaCT) study

**DOI:** 10.1186/s41747-018-0039-4

**Published:** 2018-04-27

**Authors:** Nienke G. Eijsvoogel, Babs M. F. Hendriks, Hugo B. Park, Sibel Altintas, Casper Mihl, Barbora Horehledova, Bastiaan L. J. H. Kietselaer, Harry J. G. M. Crijns, Joachim E. Wildberger, Marco Das

**Affiliations:** 1 Department of Radiology and Nuclear Medicine, Maastricht University Medical Centre, P. Debyelaan 25, PO Box 5800, 6202 AZ Maastricht, The Netherlands; 2 CARIM School for Cardiovascular Diseases, Maastricht University Medical Centre, Maastricht, The Netherlands; 3 Biomedical Sciences, Faculty of Health, Medicine and Life Sciences, Maastricht University, Maastricht, The Netherlands; 4 Department of Cardiology, Maastricht University Medical Centre, P. Debyelaan 25, PO Box 5800, 6202 AZ Maastricht, The Netherlands; 5 Department of Interventional and Diagnostic Radiology, Helios Kliniken Duisburg, Duisburg, Germany

**Keywords:** Cardiac diseases, Computed tomography, Coronary artery disease, Incidental findings, Thorax

## Abstract

Modern high-performance computed tomography (CT) scanners with improved scan acquisition times now allow for routine assessment of cardiac pathologies on chest CTs, which can result in numerous incidental cardiac findings. The CaPaCT study, an observer blinded, single-centre study, aims to assess the visibility, management and possible clinical impact of incidental cardiac pathologies that are now becoming visible on standard chest CTs. A total of 217 consecutive patients referred for a chest CT on a high-performance third-generation dual-source CT scanner will be included. Tube voltage settings will be chosen via automated kV selection. Dedicated cardiac reconstructions will be added to the standard post-processing: 0.6-mm slice thickness, 0.4-mm increment and Bv36 kernel (iterative reconstruction/strength 3). Primary endpoints will be the presence and extent of coronary artery disease (CAD) assessed via a 17-segment model. These data will be collected and analysed by two experienced, blinded cardiac radiologists. Furthermore, information on aortic and mitral valve morphology/calcification and pericardial abnormalities will be collected. The CAD Reporting and Data System classification will subsequently be used to assess the management and possible clinical burden of any incidentally detected CAD. Additionally, objective and subjective image quality (attenuation, contrast-to-noise, signal-to-noise and 5-point Likert scale) of the obtained cardiac reconstructions will be assessed.

## Key points

- Technical developments facilitate cardiac assessment on chest computed tomography (CT)
- Cardiac assessment on chest CT results in numerous incidental cardiac findings
- Cardiac assessment without clinical context has the potential to increase overdiagnosis
- Cardiac assessment without clinical context could increase patient management burden

## Background

A known disadvantage of constantly improving diagnostic imaging is the detection of incidental findings. Computed tomography (CT) technology is ever improving in both temporal and spatial resolution. Additionally, recently developed advanced reconstruction algorithms and dedicated post-processing software reduce image noise, thereby improving image quality. Equipped with these advancements, modern CT scanners accomplish well-defined imaging of cardiovascular structures. Where previously cardiac pathologies required dedicated cardiac examination protocols, these technical improvements enable detailed cardiac assessment on routine standard (not electrocardiographically triggered) chest CTs [1]. Now that it is becoming viable for thoracic radiologists to assess the heart and coronary arteries on a standard chest CT, the result could be an extensive increase of incidental cardiac findings. Herein lies the potential for large-scale reporting of incidental cardiac findings and, consequently, a huge influx of new ‘patients’ for downstream testing and potential overtreatment.

Previous research investigated the prevalence of incidental cardiac findings on chest CTs. In these studies, more than half (52–66%) of all chest CTs showed at least one incidental finding. The most frequent incidental cardiac finding was coronary artery disease (CAD), with a large number of unreported (incidental) CAD (51–80%) [2, 3]. Considering that these numbers were found on scanners equipped with outdated technology (from 4- to 64-slice scanners), the rate of incidental findings on the newest high-end scanners may be even higher. The proportion of assessable coronary arteries on chest CTs will foreseeably follow the same path, since CT techniques and image quality will continue to improve. In a few years, it is likely that current state-of-the-art scanner technology will become available on a broader scale, meaning that more medical centres could report incidental cardiac findings based on standard chest CTs. In 2006, approximately 12 million chest CT examinations were performed in the USA [4, 5]. Since then, the use of CT has continued to increase. The clinical impact of the increased patient inflow into cardiologists’ daily practice would be immense. However, despite the increasing demand, clinical studies reviewing the possible impact of these cardiac findings on patient care and on daily clinical practice are currently lacking.

Recently, a reporting and data system for CAD on CT scans was developed [6]. This new CAD-Reporting And Data System (CAD-RADS) provides a guideline on the management of patients with different CAD severities, added to which, different scoring systems have been developed to help identify patients at risk of cardiovascular events [7–9]. These, together with the CAD-RADS, could be used to assess the severity of incidental cardiac findings and determine how best to manage the care of these patients.

We can no longer overlook the heart and coronary arteries on routine chest CT. Analysis of routine chest CTs should include careful assessment of all depicted thoracic organs. Previously, a comparable imaging issue with specific and unspecific lung nodules on cardiac CT angiography led to the development of guidelines for clinical management in daily routine [10]. The *Cardiac Pathologies on standard chest CT* (CaPaCT) study has been devised to evaluate the visibility and management of incidental cardiac pathologies on an ultra-high pitch chest CT, with the help of CAD-RADS classification. The role and methods of the CaPaCT study are described here.

## Methods

### Study design and patient population

The CaPaCT study (NCT02904239) is an observer blinded, single-centre study to assess the visibility, management and possible clinical impact of CAD on standard chest CT scans, using the CAD-RADS classification. The study design was approved by the local ethical committee and institutional review board and complies with the ethical guidelines of the 1975 Declaration of Helsinki. A waiver of written informed consent was obtained from the local ethical committee.

All patients referred for a standard contrast-enhanced not electrocardiographically triggered chest CT are eligible for inclusion. Exclusion criteria consist of the standard exclusion criteria for CT scanning in the radiology department of the Maastricht University Medical Centre (MUCMC), namely pregnancy, renal insufficiency (defined as glomerular filtration rate < 30 ml/min), patients with previous severe adverse reaction to contrast material (CM), i.e. hypointensive shock, respiratory arrest and/or coinvulsions [11], and age below 18 years (Table 1). Electronic patient dossiers will be checked to see whether patients object to the use of their medical data in medical research.

**Table 1:** Inclusion and exclusion criteria for the CaPaCT study

Inclusion criteria	Exclusion criteria
Scheduled for a standard non-ECG gated ultra-high pitch thoracic CT scan	Pregnancy
≥ 18 years old	Renal insufficiency (GFR < 30 ml/min)
	Severe adverse reaction to CM
	< 18 years old
	Objection to the use of medical data stated in the EPD

*CM* contrast media, *ECG* electrocardiogram, *GFR* glomerular filtration rate, *EPD* electronic patient dossier

### Sample size calculation

Previous studies have shown that a prevalence of CAD on CT scans in asymptomatic patients increases greatly with age. Prevalence of any calcifications in asymptomatic patients varies from 44% to 54% [12, 13] ; however, no distinction is made between minimal, moderate or severe CAD. Kelkar et al. [12] reported a prevalence of moderate to severe CAD of 17% in a population of 2363 asymptomatic patients with a low-intermediate Framingham risk score. This prevalence of 17% was used for the sample size calculation, as it includes the clinically relevant CAD, which requires downstream testing and/or treatment. Given a 95% confidence interval (12–22%), a total of 217 patients and scans are needed to reach a 17% prevalence of moderate to severe CAD in our population.

### Study endpoints

The primary endpoints of the CaPaCT study are the prevalence and severity of incidental CAD on standard chest CTs. Presence of CAD is defined as the presence of calcified or soft plaques with luminal narrowing in the coronary arteries. Management of patients with CAD will be assessed with help of the CAD-RADS classification (see below evaluation of pathologies). Furthermore, information on other cardiac pathologies will be collected (see below data analysis). Considering that technical advancements are an essential part of the increasing CAD detectability on chest CT scans, secondary endpoints will include the subjective and objective image quality of utilised standard chest CTs, in combination with radiation dose and administered contrast material volume. Thirdly, this study will investigate the magnitude and effect of this newly diagnosed, mostly asymptomatic group of patients on clinical practice.

### CT protocol

All scans will be performed on a high-end third-generation dual-source CT scanner (Somatom Force, Siemens Healthineers, Forchheim, Germany). The scans will be standard, not electrocardiographically gated, ultra-high pitch chest CT acquisitions. A 2 × 192 × 0.6 mm slice collimation and gantry rotation time of 0.25 s will be used. A dynamically adapted pitch value of 2.65 to 3.00 will be used to increase the scan field of view where needed (354–391 mm). The tube voltage will be set by automated tube voltage selection software (automated tube voltage selection, CAREkV, Siemens Healthineers), with a quality reference tube voltage of 110 kVp_qual.ref_ and reference tube current of 40 mAs_qual.ref_ (CareDose 4D™, Siemens Healthineers). Scan delay will be determined with the bolus tracking technique, wherein a circular region of interest will be placed in the ascending aorta; a threshold of 50 Hounsfield units and an additional delay of 6 s (table movement and breath hold command) will be used to start scanning.

A dedicated cardiac reconstruction will be added to the standard (thoracic) post-processing workflow, namely images will be reconstructed with a 0.6 mm slice thickness, an increment of 0.4 mm and a Bv36 kernel (Advanced Modelled Iterative Reconstruction, ADMIRE, strength 3). Dose monitoring software (Radimetrics Enterprise Platform; Bayer Healthcare, Berlin, Germany) will be used to record all dose-related parameters (e.g. mAs_eff_, CT dose index volume, dose-length product and mSv).

### Contrast injection protocol

All patients will receive pre-warmed (37 °C, 99 °F) iodinated contrast material (Ultravist; 300 mg I/ml, Bayer Healthcare, Berlin, Germany), administered automatically using a dual-head CT power injector (Stellant, Bayer). The CM injection protocols will be adapted by varying the iodine delivery rate according to the kV setting chosen by ATVS (Table 2). Eighteen, 20 or 22 gauge needles will be inserted in the antecubital vein. CM monitoring software (Certegra^TM^ Informatics Solution, Bayer) will be used to record all relevant CM injection parameters (e.g. volume, flow rate, peak flow rate, peak pressure and total iodine load) for each patient.

**Table 2:** Contrast injection protocols for different kV settings as chosen by CAREkV

Parameter	120 kV	110 kV	100 kV	90 kV	80 kV	70 kV
Main bolus 100% CM, ml	44	40	36	33	30	27
Mixed bolus (50% CM/50% NaCl), ml	36	33	30	27	25	22
Saline flush, ml	20	20	20	20	20	20
Flowrate, ml/s	5.1	4.7	4.2	3.9	3.5	3.2
Iodine delivery rate, gI/s	1.5	1.4	1.3	1.2	1.1	0.9

*CM* contrast material, *kV* tube voltage

### Data analysis

All CT images will be anonymised before analysis. The images will be assessed using axial slices and multiplanar reconstruction with dedicated software (Syngo.Via™, Siemens). Centrelines will be drawn by an experienced researcher in the coronary arteries, including the right coronary artery, left main, left anterior descending artery and circumflex artery, prior to pathology assessment. Two independent, experienced cardiac radiologists will read the images, blinded to one another’s results as well as patient characteristics. Information about CAD, aortic valve morphology, aortic valve calcifications, mitral valve calcifications and pericardial abnormalities (e.g. effusion or calcifications) will be collected and analysed. The CAD severity will be assessed according to a modified 17-segment model from the American Heart Association [14]. Plaques shall be categorised according to the CAD-RADS classification [6]. Electronic patient dossiers (SAP 7.3, SAP SE, Walldorf, Germany) will be consulted to see whether or not these findings are previously mentioned in the patient’s history or on prior chest CTs.

Objective image quality will be assessed by intravascular attenuation (Hounsfield units), contrast-to-noise ratio and signal-to-noise ratio. Image noise will be defined as the standard deviation of the vessel attenuation. Contrast-to-noise ratio will be stated as intravascular attenuation minus epicardial fat attenuation, divided by the standard deviation of the epicardial fat attenuation. Signal-to-noise ratio will be stated as intravascular attenuation divided by the standard deviation of the intravascular attenuation [15–17]. Intravascular and epicardial fat attenuation will be measured via circular regions of interests in the vessels and epicardial fat, carefully avoiding vessel walls and atherosclerotic plaques. Subjective image quality in terms of motion artefacts will be evaluated using a 5-point Likert scale (5 = excellent, no motion artefacts; 4 = good, minor motion artefacts; 3 = sufficient, moderate motion artefacts; 2 = moderate, significant motion artefacts but diagnostic; 1 = non-diagnostic due to motion artefacts).

### Statistical analysis

Continuous variables will be stated as mean and standard deviation. Categorical variables will be compared by using a χ^2^ test and stated as percentages. A linearly weighted κ will be calculated for the interobserver agreement on the assessment of CAD and motion artefacts. The image quality of the scans with optimised contrast injection protocols will be assessed. Continuous variables of different kV groups will be compared using one-way analysis of variance. In case the data is not normally distributed, non-parametric tests will be used to compare the means of different kV groups. All *p* values will be two-sided and a *p* value lower than 0.05 will be considered statistically significant.

## Discussion

The importance and clinical impact of assessment of the heart and coronary arteries is becoming clearer with the help of current evidence; cardiovascular disease is the leading cause of death worldwide and CAD accounts for up to 20% of all deaths in Europe annually [18]. The need for detection of CAD in patients with diabetes mellitus or undergoing non-cardiac surgery and in cancer treatment decision-making has been demonstrated [19–25]. The purpose for a contrast-enhanced chest CT is often associated with cancer screening or follow-up. Thus, knowledge of the presence and extent of CAD in these patients is required for optimal treatment.

Other studies that assess patients at risk for future cardiovascular events with help of incidental cardiac findings on chest CT have been conducted. Jairam et al. [8] developed a risk score calculator to evaluate patients at high risk for a cardiovascular event. In contrast to the 64-slice multidetector CT used for that study, the CaPaCT study will use a modern third-generation dual-source CT scanner. Further, these studies did not assess the medical management or clinical impact of these additional patients. Moreover, none of these studies implemented a cardiac reconstruction and coronary centrelines in their scan protocol and no studies used the newly developed CAD-RADS classification. The CaPaCT study will assess the occurrence of incidental CAD in patients undergoing chest CT and how radiologist should report on this. It is hoped that the CaPaCT study will provide insight into the magnitude of this inevitable escalation of possibly asymptomatic cardiovascular patients.

## Abbreviations

CAD: coronary artery disease
CAD-RADS: Coronary Artery Disease Reporting and Data System
CaPaCT: Cardiac Pathologies on standard chest CT
CM: Contrast material
CT: computed tomography
